# Family Planning in the Context of Latin America's Universal Health Coverage Agenda

**DOI:** 10.9745/GHSP-D-17-00057

**Published:** 2017-09-27

**Authors:** Thomas Fagan, Arin Dutta, James Rosen, Agathe Olivetti, Kate Klein

**Affiliations:** a Palladium, Health Policy Plus, Washington, DC, USA.; b Avenir Health, Health Policy Plus, Washington, DC, USA.

## Abstract

**Background:**

Countries in Latin America and the Caribbean (LAC) have substantially improved access to family planning over the past 50 years. Many have also recently adopted explicit declarations of universal rights to health and universal health coverage (UHC) and have begun implementing UHC-oriented health financing schemes. These schemes will have important implications for the sustainability and further growth of family planning programs throughout the region.

**Methods:**

We examined the status of contraceptive methods in major health delivery and financing schemes in 9 LAC countries. Using a set of 37 indicators on family planning coverage, family planning financing, health financing, and family planning inclusion in UHC-oriented schemes, we conducted a desk review of secondary sources, including population surveys, health financing assessments, insurance enrollment reports, and unit cost estimates, and interviewed in-country experts.

**Findings:** Although the modern contraceptive prevalence rate (mCPR) has continued to increase in the majority of LAC countries, substantial disparities in access for marginalized groups remain. On average, mCPR is 20% lower among indigenous women than the general population, 5% lower among uninsured women than insured, and 7% lower among the poorest women than the wealthiest. Among the poorest quintile of women, insured women had an mCPR 16.5 percentage points higher than that of uninsured women, suggesting that expansion of insurance coverage is associated with increased family planning access and use. In the high- and upper-middle-income countries we reviewed, all modern contraceptive methods are typically available through the social health insurance schemes that cover a majority of the population. However, in low- and lower-middle-income countries, despite free provision of most family planning services in public health facilities, stock-outs and implicit rationing present substantial barriers that prevent clients from accessing their preferred method or force them to pay out of pocket.

**Conclusion:**

Leveraging UHC-oriented schemes to sustain and further increase family planning progress will require that governments take deliberate steps to (1) target poor and informal sector populations, (2) include family planning in benefits packages, (3) ensure sufficient financing for family planning, and (4) reduce nonfinancial barriers to access. Through these steps, countries can increase financial protection for family planning and better ensure the right to health of poor and marginalized populations.

## Abstract (precis)

Latin American countries have expanded family planning along with universal health coverage (UHC). Leveraging UHC-oriented schemes to increase family planning program coverage, equity, and financing requires:

- Prioritizing poor and indigenous populations
- Including family planning services in all benefits packages
- Ensuring sufficient supply of commodities and human resources to avoid stock-outs and implicit rationing
- Reducing nonfinancial barriers to access

## INTRODUCTION

Over the past 50 years, countries in Latin America and the Caribbean (LAC) have successfully expanded access to family planning services. The modern contraceptive prevalence rate (mCPR) has increased in every country in the LAC region, and by more than 30 percentage points in countries such as El Salvador, Honduras, Nicaragua, Paraguay, and Peru.^1^ For 2015, the mCPR for the region is estimated at 66.7%, and unmet need for family planning is estimated at 10.7%.^2^ The region has seen corresponding decreases in the total fertility rate, which has fallen from 5–6 children per woman to just 2.2, nearing the replacement fertility level of 2.1. This decline was driven by improvements in access to family planning services, such as the subsidized provision of contraceptives through social marketing and the inclusion of family planning services in the basic package of services provided by government-supported health facilities. Over this period, support from international organizations and donors, including the United States Agency for International Development (USAID), the International Planned Parenthood Federation, and the United Nations Population Fund (UNFPA), was critical in strengthening family planning programs. Improvements in education, income levels, and infant and child mortality rates have further contributed to the decline in the total fertility rate.^1^

Access to family planning in Latin America has resulted in increased use of modern methods and decreases in the total fertility rate.

The success of family planning programs in many LAC countries did not occur overnight, but has rather been part of a steady process of development of countries' health systems as they advance toward universal health coverage (UHC). In recent decades, many LAC countries have recognized health as a fundamental—and often constitutional—right of their citizens and embraced the goals of UHC.^3^ (A review of this process in the LAC region is beyond the scope of this paper.) UHC implies an assurance of financial protection against the cost of health care (i.e., the ability to access services without facing financial hardship) and provision of quality services across a full range of promotive, preventive, curative, rehabilitative, and palliative interventions, for all people.^4^ Such services can be provided through any variety of health financing and delivery channels, including social or private health insurance and public or private facilities.

As part of the movement toward UHC, many countries in the LAC region made efforts to reform their health financing to increase the pooling of funds, guaranteed access to an essential package of services, and financial protection from the cost of health services for the vulnerable. These reforms were based on other, longer-term efforts in primary health care strengthening, regulation, and social mobilization. Broadly, LAC countries have used 3 types of financing mechanisms—which this paper henceforth refers to as UHC-oriented schemes—as the means to further these goals:

- Social health insurance
- Private health insurance
- Public tax-funded provision

Social health insurance (SHI) schemes have been a primary mechanism for increasing health insurance coverage in many LAC countries. These schemes are state-supported and are typically financed through payroll contributions from formal-sector workers. In addition to these “contributory” schemes, high- and upper-middle-income countries in the region have expanded SHI to include “subsidized” schemes, with the state subsidizing enrollment for informal workers and the poor.^5^ Such subsidized schemes ensure greater financial protection and a higher quality of services for poor and vulnerable populations.^5^ On occasion, governments have set up and substantially financed autonomous insurance schemes focused on the poor, for example in Peru. Henceforth, this paper refers to these as “other government-supported schemes.” Private health insurance (PHI) plays only a small role in health financing in LAC countries and covers mainly higher-income individuals. These PHI schemes can be stand-alone or supplementary to SHI. They are usually supported by voluntary contributions from formal-sector employees and/or their employers, or from those with otherwise higher income status.

State-supported social health insurance has been a mechanism for increasing health care coverage in the region.

Many governments continue to provide health services through public facilities, often for free. The idea of a comprehensive public health care network financed through tax revenue and provided free to all residents (similar to the National Health Service model in the United Kingdom) represents theoretically near-universal financial protection from the cost of health care. In practice in many LAC countries, particularly lower-income countries, free provision is insufficient, alone, to ensure adequate range and quality of services. These limitations to public tax-funded provision, within the context of family planning services, are discussed in this article. Additional health financing modalities may, therefore, be needed to cover all population groups and sufficiently meet the goals of UHC.

In practice, free provision of health services at public facilities is not enough to ensure people's access to a sufficient range of services.

SHI is the primary mechanism for health sector financing in much of the LAC region, and the term “social health insurance” is often used interchangeably with the term “universal health coverage.” However, it is important to note that SHI arose separately from and before broad, UHC-oriented efforts. Nascent SHI schemes in LAC began with a focus on hospital care, curative interventions, and protection against catastrophic health care expenditures.^6^ Only with the recent adoption of explicit UHC-oriented goals has the focus begun to shift to include primary and preventive services, including family planning.

Before their SHI schemes matured, some countries' family planning programs benefited from high levels of sustained external support, primarily from USAID.^1^ Improvements in family planning access over the 1990s and 2000s in many LAC countries were due to a variety of factors and not only the expansion of UHC-oriented schemes.^1^^,^^7^ However, integration of family planning into UHC-oriented efforts has been suggested as a possible way to continue the improvement in family planning access and use^8^ as well as to provide sustainable family planning financing in countries with high mCPR, especially where external financing has tapered off (this is referred to as “graduation”).

Theoretically, UHC-oriented schemes, including SHI, should promote financial protection in the context of the cost of family planning—if the related benefits are included in the scheme—by increasing prepayment and by subsidizing fully or partially the cost of health services, including premium payments, for some groups. This can help ensure effective family planning access for poor and vulnerable women, including those from indigenous groups. Examining recent evidence can help to determine if this has been the case in countries with mature family planning and UHC programs functioning in parallel, as well as to discern lessons from and for other countries on the path to UHC.

Theoretically, UHC-oriented schemes can help ensure that poor and marginalized women, including indigenous women, have access to family planning.

Many LAC countries, especially in Central America and the Caribbean, have recently graduated from or are currently facing graduation from external support for family planning. There is thus a need to consider long-term plans for sustainably financing the scale-up of family planning access and use. For these countries, learning about the recent links between family planning access and UHC-oriented health insurance schemes in the region becomes critical.

However, despite extensive literature on both UHC and family planning progress in LAC countries, as well as on the recent family planning graduation of some LAC countries, little analysis exists to link ongoing family planning efforts to the broader LAC UHC agenda. The purpose of our analysis is therefore to better understand the relationship between the coverage of UHC-oriented schemes and family planning. To our knowledge, this is the first effort to systematically examine the status of family planning within UHC schemes. Including family planning within UHC-oriented schemes involves not only legal status (i.e., the inclusion of family planning in the benefits package), but also the consistent availability of family planning both within and across facilities, the availability of a range of methods, and appropriate co-pays (i.e., co-pays that do not expose clients to financial hardship) for accessing contraceptive methods.

The purpose of this analysis is to understand the relationship between universal health care coverage and family planning.

By considering family planning in the context of UHC, LAC and other countries may make more informed decisions on how to integrate family planning into UHC-oriented schemes. Our analysis examines the following key questions for a selected sample of countries in the LAC region:

1. What is the current level of coverage for SHI and other risk-pooling mechanisms, and how does this vary by geography, ethnicity, and income level? What variation exists between countries in their progress toward UHC, and toward universal family planning access as a subset of UHC?
2. What is the status of family planning services within major insurance and health financing schemes? Where family planning services are included, what is the actual coverage and level of access (e.g., methods and availability)? What is the level of financial protection offered for family planning within these schemes (i.e., are co-pays required)?
3. What drives variation in the status of family planning services within insurance schemes both within and across LAC countries? What lessons can be learned from the linkages of family planning and reproductive health and UHC reforms in the region, especially to inform policies in other countries in the region with lagging family planning indicators? What broad lessons can be learned as implications for countries in other regions?

## METHODS

We purposively selected and analyzed 9 countries throughout the LAC region—3 each from South America, Central America, and the Caribbean. These countries were selected to exhibit a range of income levels, UHC-related progress, and family planning access. For each country, we collected data using a standardized set of 37 indicators across 4 key areas: family planning coverage; family planning financing; health financing; and family planning inclusion in UHC-oriented schemes. A full list of the indicators used is available in Supplement 1.

The team performed a desk review of existing literature on family planning and health financing in the countries of interest, and in LAC broadly. Key data sources included population-level surveys, primarily Demographic and Health Surveys (DHS) and Multiple Indicator Cluster Surveys; health financing assessments; and insurance enrollment reports. DHS and other survey data were further cross-tabulated to examine inequalities in access based on socioeconomic and insurance status, ethnicity, geography, and other factors.

The team also conducted key informant interviews with in-country experts in family planning. These interviews informed our assessment of the current status and inclusion of contraceptive methods within public health service provision and SHI and PHI schemes. The team used additional data, including estimates of out-of-pocket (OOP) spending on family planning provided by the Netherlands Interdisciplinary Demographic Institute, to conduct its own estimates of total family planning expenditures. Methods for these estimates are discussed in Supplement 2.

Interviews with in-country experts informed our assessment of how family planning is included in current health insurance schemes.

## FINDINGS

The following section summarizes our key findings on family planning access, UHC progress, and the inclusion of family planning in UHC-oriented schemes for each of the study countries.

### Chile

**FIGURE 1:**
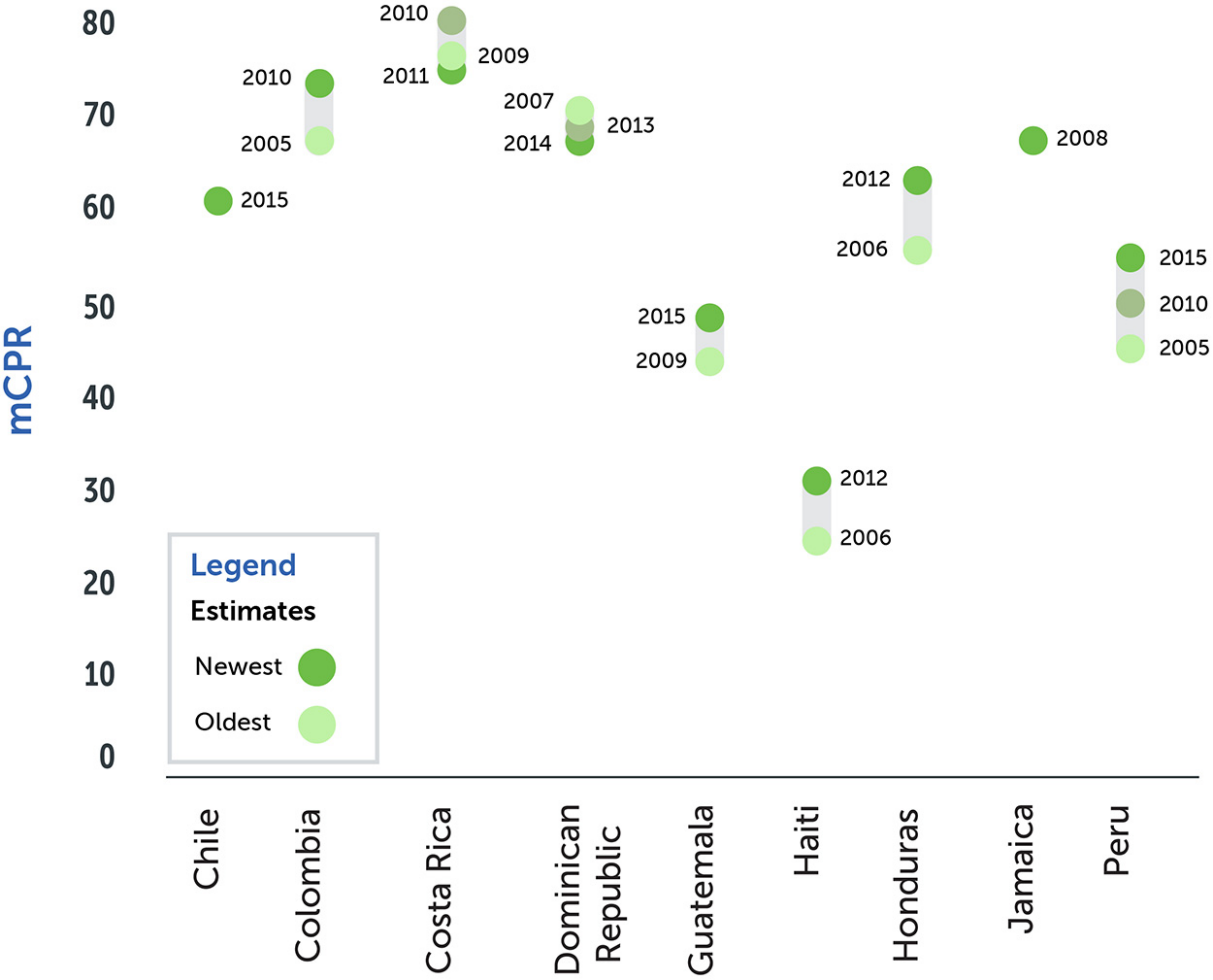
mCPR by Country Among Women Married or in Union (2005–2015) Abbreviation: mCPR, modern contraceptive prevalence rate.

**FIGURE 2:**
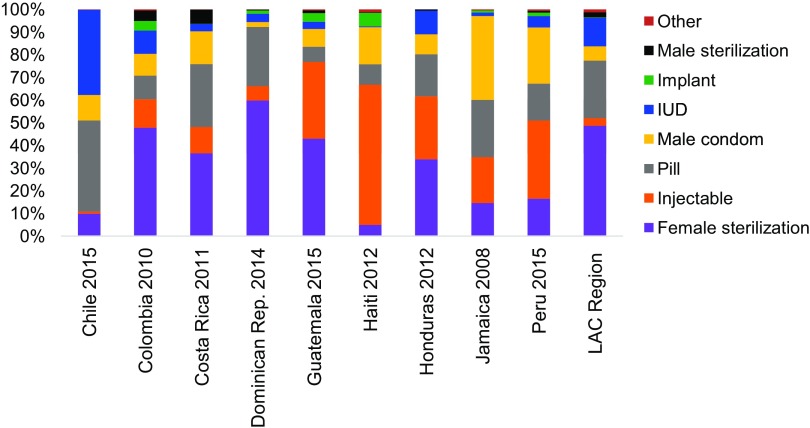
Contraceptive Method Mix by Country Among Contraceptive Users Married or in Union (2008–2015) Abbreviations: IUD, intrauterine device; LAC, Latin America and the Caribbean.

Chile is the only high-income country in our sample. As of 2013, Chile had a total fertility rate of 1.79, which is below both replacement level and the level of many other economically developed countries.^9^ However, family planning uptake in Chile has been relatively slow, with mCPR increasing from 43% in the mid-1980s to only an estimated 62% by 2016 (Figure 1), while unmet need is still at 13% (2016)—above the regional average of 10.7%.^10^ Of modern methods, the pill and intrauterine devices (IUDs) are the most popular, accounting for 40.2% and 37.5% of overall use, respectively (Figure 2).^10^ Chile has relatively little disparity in access to family planning services along lines of geography or wealth.

Chile's health insurance expansion has achieved near-universal coverage through the country's UHC-oriented scheme, Fondo Nacional de Salud (FONASA), which covers 76% of the population, and Instituciones de Salud Previsional (ISAPRES), which is privately financed (primarily by employers) and covers 17% of the population (Table). Beneficiaries of both schemes have access to services that address 80 priority health conditions.^22^ FONASA covers all major family planning methods, and all FONASA primary care clinics provide family planning free of charge, with the exception of sterilization. Wealthier beneficiaries have a co-pay for sterilization, ranging from approximately $20 to $50, based on income group.^23^ Under ISAPRES, coverage of contraceptive methods and associated co-pays varies greatly by plan, with some plans providing family planning with small or no co-pays while others requiring clients to pay fully OOP for family planning. Additionally, many clients choose to pay OOP for pills and condoms in pharmacies. Experts identified wait times in FONASA facilities as another reason for OOP payments, despite broad insurance coverage (personal communication with Eduardo Soto Fernandez, Nurse-Midwife Advisor, National Women's Health Program, Life Cycle Department, Ministry of Health, August 2016).

Despite near-universal insurance coverage, Chile has a lower mCPR than many countries in the region.

**TABLE.** Estimated Health Insurance Coverage by Major Insurance Schemes, Selected Latin American and Caribbean Countries

	SHI and/or Other^a^	PHI	Total^b^
Chile (2015)^11^	77%	17%	94%
Colombia (2015)^12^	97%	6%	97%
Costa Rica (2013)^13^	94%	—	94%
Dominican Republic (2013)^14^	28%	29%	57%
Guatemala (2014)^15^^,^^16^	18%	5%	23%
Haiti (2014)^17^	3%	4%	7%
Honduras (2015)^18^	19%	3%	19%
Jamaica (2013)^19^^,^^20^	19%^c^	19%	<38%
Peru (2015)^21^	73%	2%	73%

Abbreviations: PHI, private health insurance; SHI, social health insurance.

^a^ Aggregate of SHI and other government-supported insurance schemes, as applicable.

^b^ Total insurance coverage may be lower than the sum of SHI and PHI coverage, due to overlap of covered populations.

^c^ National Health Fund coverage only—limited benefits and not SHI.

Given Chile's existing near-universal insurance access, further increases in mCPR will require addressing programmatic challenges to family planning, including reducing nonfinancial barriers to sterilization and making services more responsive to groups such as adolescents (personal communication with Eduardo Soto Fernandez, Nurse-Midwife Advisor, National Women's Health Program, Life Cycle Department, Ministry of Health, August 2016).

### Colombia

Colombia reached replacement-level fertility by 2010. Female sterilization accounts for nearly half of the method mix (Figure 2) behind the country's high mCPR (73%) (Figure 1).^24^ Landmark legislation in 1993 established what is now a near-universal 2-tiered compulsory SHI scheme. A contributory scheme for formal-sector workers, funded by payroll taxes, covered 43% of the population in 2015; a subsidized scheme for the poor, funded through general taxation and other sources, covered 49% of the population.^25^

Colombia's 2-tiered insurance scheme, which includes a contributory and a subsidized program, covers more than 90% of the population.

**FIGURE 3:**
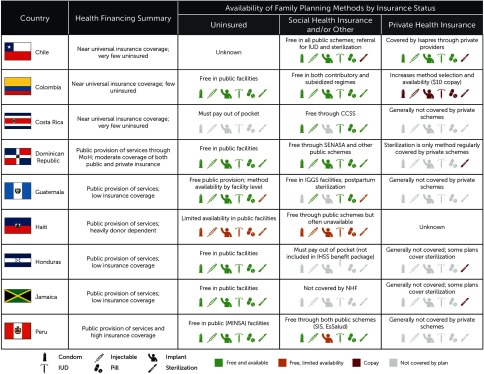
Availability of Family Planning Methods by Country and Insurance Status Abbreviations: CCSS, Caja Costarricense de Seguridad Social (national SHI scheme); IGSS, Instituto Guatemalteco de Seguridad Social (national SHI scheme); IHSS, Instituto Hondureño de Seguridad Social (national SHI scheme); IUD, intrauterine device; OOP, out of pocket; PHI, private health insurance; SeNaSa, Seguro Nacional de Salud; SHI, social health insurance.

Both the contributory and subsidized schemes cover the same minimum package of health services, including all major family planning methods, free of charge (Figure 3).^26^ However, in practice, many health facilities—particularly in remote areas of the country—lack the full range of methods (personal communication with Nora Quesada, Regional Director for Latin America and the Caribbean, John Snow, Inc., August 2016). Rural areas lack diversity of providers, and hence many members of subsidized schemes rely on public facilities alone and have otherwise more limited ability to choose providers.^27^ Moreover, a substantial number of facilities lack personnel trained in providing all family planning methods.^28^ Wait times for sterilization can be up to 3 months, far in excess of the 5-day maximum mandated by the Ministry of Health and Social Protection.^28^ Colombians who can afford it may obtain supplementary insurance through a prepaid plan that allows broader choice in providers and minimal wait times for surgeries, including sterilization.

Despite high insurance coverage, OOP spending on family planning is estimated to be substantial in Colombia (personal communication, Karin Vrijburg, Researcher, NIDI, and Erik Beekink, Project leader, UNFPA-NIDI Resource Flows Project on Family Planning, NIDI, June, 2016). In addition, equity in access to family planning services remains an issue as unmet need for family planning is almost twice as high among the poorest women as among the wealthiest.^29^

Despite high insurance coverage in Colombia, there is substantial out-of-pocket spending on family planning.

### Costa Rica

**FIGURE 4:**
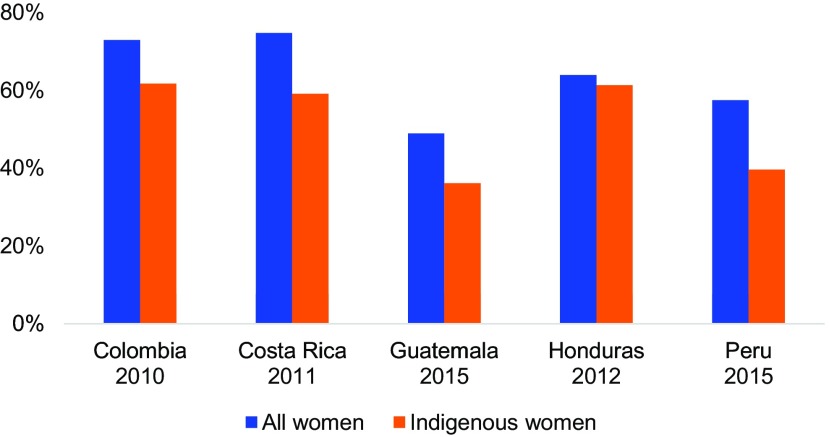
mCPR in 5 Countries by Indigenous Status Among Women Married or in Union (2010–2015) Abbreviation: mCPR, modern contraceptive prevalence rate.

Costa Rica has achieved substantial improvements in access to family planning, with among the highest mCPRs in the region—74.7% (Figure 1).^30^ However, poor and indigenous Costa Ricans have lower mCPR—and, conversely, higher unmet need—than the general population (Figure 4). The country's national SHI scheme, the Caja Costarricense de Seguridad Social (CCSS), covers 94% of the population.^13^ While CCSS allows the poor to enroll free of charge, in 2013 CCSS began to deny services to unenrolled individuals unless they pay OOP or their life is in danger.^31^

CCSS, which was first founded in 1941, assumed responsibility for all public health service provision, including family planning, in the early 1990s.^32^^,^^33^ CCSS provides the 6 most common contraceptive methods—female and male sterilization, oral and injectable contraception, IUDs, and male condoms^34^—free of charge to enrollees (Figure 3). The high level of coverage and the status of family planning under CCSS suggests that Costa Rica's high mCPR has been closely tied to CCSS's expansion. However, financial challenges and higher demand for CCSS services have put a strain on service delivery and increased wait times, particularly for specialized and inpatient services.^35^ Such wait times may help to explain why, despite near-universal coverage under CCSS, an estimated one-quarter of family planning clients obtain family planning services in the private sector (personal communication, Karin Vrijburg, Researcher, NIDI, and Erik Beekink, Project leader, UNFPA-NIDI Resource Flows Project on Family Planning, NIDI, June, 2016). Costa Ricans who must pay OOP tend to face higher prices for family planning services than in other LAC countries (personal communication, Karin Vrijburg, Researcher, NIDI, and Erik Beekink, Project leader, UNFPA-NIDI Resource Flows Project on Family Planning, NIDI, June, 2016), and we estimate that the one-quarter of family planning clients who access services in the private sector account for 62% of family planning spending in Costa Rica.

Despite the legal status of family planning as a covered benefit under CCSS, guaranteeing the timely availability of a full range of methods will be necessary to ensure full financial protection for family planning, and the financial stability of CCSS will be critical to sustaining the high level of family planning use that Costa Rica has already achieved.

Procurement and distribution of a full range of methods is necessary to ensure access to family planning, even in the context of near-universal insurance coverage.

### Dominican Republic

Over 40 years, the Dominican Republic achieved a more than 3-fold increase in mCPR, which peaked at 70% in 2007 (Figure 1).^36^ Since USAID graduation in 2009, contraceptive prevalence has plateaued and integration of family planning into the country's major health financing mechanisms has achieved mixed results. Insurance coverage, which more than doubled between 2007 and 2013, is divided roughly equally between public and private providers, at 28% and 29% of the population, respectively.^14^ The primary public scheme, the Seguro Nacional de Salud (SeNaSa), provides the 6 most common contraceptive methods free of charge. It is dependent on the Ministry of Public Health for commodities, however, and the ministry itself provides family planning services free of charge. Private insurers, which play a major role in health financing, generally fail to cover family planning, with the exception of postpartum sterilization (personal communication with Sonia Anderson, Country Director, Capacity Plus, June 2016).

**FIGURE 5:**
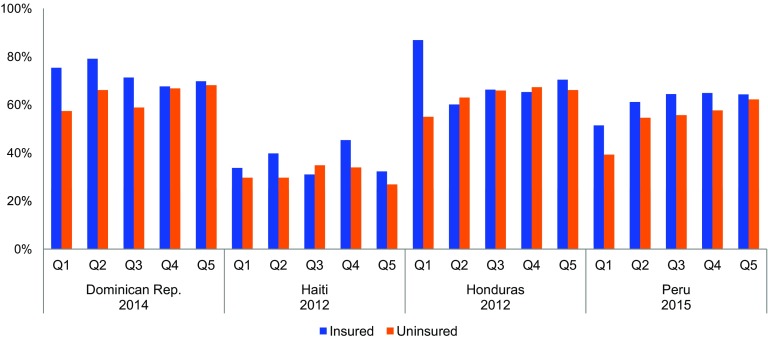
mCPR in 4 Countries by Insurance Status and Wealth Quintile (Q1=Poorest, Q5=Wealthiest) Among Women Married or in Union (2012–2015) Abbreviation: mCPR, modern contraceptive prevalence rate.

In the Dominican Republic, insured women in the poorest 2 wealth quintiles have the highest mCPR (77%), in part due to SeNaSa's high coverage among poor women (Figure 5).^37^ Forty-one percent of women in the poorest quintile are covered by SeNaSa, compared with 14% in the wealthiest quintile.^14^ On the other hand, mCPR does not vary much with insurance status among the wealthiest 2 quintiles, likely due to the exclusion of family planning from private insurance benefits packages. Poor, uninsured women remain particularly vulnerable and face the lowest mCPR. Expanding financial protection for health care to these women, while ensuring the inclusion of family planning in benefits packages for women currently insured, will be critical for the Dominican Republic to reach universal access to family planning services.

In the Dominican Republic, modern contraceptive prevalence is highest among poor women covered by state-supported insurance.

### Guatemala

Family planning access in Guatemala continues to be plagued by stark inequalities and systemic weaknesses in the health sector. In Guatemala, the mCPR is only 48.9% (Figure 1), and it is substantially lower for rural, indigenous (Figure 4), and young women, at 43.2%, 36.1%, and 31.3%, respectively.^38^ Use of traditional methods is particularly high, at 19% of total contraceptive use, and is highest among indigenous women, at 28%. Both cultural and programmatic factors create barriers to family planning access. In particular, the cancellation of the Extension of Coverage Program in 2014 left many in rural areas—nearly 30% of the country's population—without access to basic health services, including family planning.^16^^,^^39^

Currently, an estimated 60% of family planning services in Guatemala are provided in public facilities (personal communication, Karin Vrijburg, Researcher, NIDI, and Erik Beekink, Project leader, UNFPA-NIDI Resource Flows Project on Family Planning, NIDI, June, 2016). The Ministry of Public Health and Social Assistance provides condoms and injectable and oral contraception at primary care facilities, and IUDs, implants, and sterilization at some secondary-level facilities. Since 2004, Guatemala has allocated 15% of taxes on alcoholic beverages to fund family planning and reproductive health; of the total amount, 30% is designated for family planning commodities. However, use of these funds for family planning is inconsistent, and stock-outs are frequent.^40^ As a result, many clients seek their method of choice in the private sector. Private insurance coverage is minimal in Guatemala, and even the country's SHI scheme, the Instituto Guatemalteco de Seguridad Social (IGSS), which covers approximately 18% of the population,^16^ plays a relatively minor role in contraceptive provision.

It is estimated that 60% of family planning services in Guatemala are provided in public facilities, yet stock-outs are common.

### Haiti

Haiti, one of the world's poorest countries, has a total fertility rate of 3.5.^41^ From 1994 to 2012, the mCPR in Haiti rose from 13.2% to 31.3% (Figure 1), driven by strong donor support, which increased access to services and strengthened the procurement system for family planning commodities. Despite this increase, Haiti has one of the highest rates of unmet need for family planning in the world, at 35%.^41^ There is minimal variation in mCPR between rural and urban areas or by geographic region, and low use of methods such as IUDs and sterilization (Figure 2) may suggest a lack of trained medical personnel in many facilities. Government health expenditures represented just 7% of total health expenditures (THE) in 2013.^42^ Thus, Haiti continues to rely heavily on donor funding (64% of THE) and OOP spending (30%). Two public institutions, Office d'Assurance Accidents du Travail, Maladie et Maternité (OFATMA) and Office National d'Assurance-Vieillesse (ONA), offer health insurance and social security: OFATMA for formal sector employees, and ONA for the elderly and disabled. Haiti also has 9 private insurance providers. Together, public and private schemes cover 7% of the population, with much lower coverage rates in rural areas (Table).

Haiti has one of the highest rates of unmet need for family planning in the world.

Despite a 2014 law mandating free contraceptive methods in all public health facilities, the lack of funding means that certain methods are often unavailable.^43^ OFATMA covers family planning services, but these services are not always available in the network of affiliated clinics.^44^ Overall, over half of family planning services are paid for OOP in the private sector. Poor financial protection for health care contributed to 3%–4% of Haitians facing catastrophic health expenditures in 2013.^45^ One promising step forward was the creation of a free health insurance card, “La Carte Rose,” in 2012 to facilitate access to high-quality health care, including obstetric and gynecologic services.^46^

While La Carte Rose may be one option for ensuring more comprehensive and reliable reproductive, maternal, newborn, and child health services, including family planning, low rates of insurance coverage and the lack of inclusion of family planning services and commodities in existing insurance schemes remain major challenges for Haiti.

### Honduras

Honduras has made substantial recent progress in family planning use, with mCPR having increased from 56% in 2006 to 64% in 2012 (Figure 1).^47^ Variations in family planning use are most pronounced between urban and rural areas, with the country's most rural region, Gracias a Dios, having an mCPR of only 49%.^47^

OOP spending is the primary source of family planning financing—composing approximately three-quarters of total family planning expenditures—and there is an important need to improve financial protection for family planning. Although public facilities provide condoms, implants, oral and injectable contraception, and sterilization services free of charge, stock-outs are a major problem, with 70% of facilities registering a stock-out of at least 1 contraceptive commodity in the past 6 months.^48^ Insurance coverage is low, with 19% of Hondurans enrolled in the national SHI scheme, the Instituto Hondureño de Seguridad Social (IHSS), and just 3% covered by PHI.^18^^,^^47^ Neither private schemes nor IHSS play a major role in family planning provision; IHSS does not formally include family planning planning in its benefits package. As a result, most clients seeking family planning services in the private sector, which provides 43% of the modern methods available, must pay OOP.

In Honduras, individuals' out-of-pocket spending composes approximately 75% of total family planning expenditures.

As Honduras has transitioned away from donor financing, with formal graduation from USAID support in 2015, there have been continued efforts to link family planning with national priorities and strategies, including the reduction of maternal mortality.^49^ Additionally, Honduras has recently adopted a plan to address unmet need for family planning in rural areas, and ongoing efforts toward decentralization may have positive impacts on family planning programs. However, there remains a need for increased family planning financing. Experts also cite a need for improvements in strategic planning for family planning at the national level (personal communication with Julio Zúniga, Country Director, Pasmo Honduras, June 2016).

### Jamaica

In Jamaica, the mCPR among women married or in union increased from 62.9% in 1997 to 68.2% in 2008 (Figure 1), while the total fertility rate among this population has fallen.^50^ Paradoxically, mCPR is highest (71%) among the poorest quintile of women and lowest (65%) among the second wealthiest quintile.^51^

Most Jamaicans depend on the public sector for subsidized services or pay OOP for health care; for most contraceptive methods, they pay well above the regional average (personal communication, Karin Vrijburg, Researcher, NIDI, and Erik Beekink, Project leader, UNFPA-NIDI Resource Flows Project on Family Planning, NIDI, June, 2016). Poor households primarily access health services in public facilities (63%), while wealthy households favor private providers (76.6%), data that highlight the importance of public health and family planning services to ensuring equity in family planning access.^52^ Among women who obtained contraceptives from government sources in 2008, just over half (51.2%) reported that family planning services were available at any time.^52^ With the exception of isolated PHI schemes that cover limited numbers of people, health insurance in Jamaica does not include family planning benefits. The country has no formal SHI program. As part of the social security program managed by the Ministry of Labour and Social Security, retired formal-sector workers can access specific benefits covering the cost of visits, services, and prescriptions, alongside co-payment. There is also a program (Jamaica Drugs for the Elderly Programme [JADEP]) that provides free medicines for 10 conditions for those over 60 years of age. The National Health Fund, established in 2003, is a statutory entity that provides a limited benefit subsidizing the cost of pharmaceuticals provided at public and private facilities for 16 chronic diseases. It now covers 19% of the population, and family planning is not a benefit.^53^ Ostensibly, primary health care services are provided for free at all government health facilities. Jamaica abolished user fees in public facilities in 2008, and this has increased use of health care, but the effects on family planning access and use are uncertain.^53^

Most health insurance in Jamaica does not cover family planning.

### Peru

In Peru, total fertility fell from 4.3 in the mid-1980s to 2.5 by 2015.^21^ At the same time, mCPR has increased rapidly, from 23% of married women in the early 1980s to 53% by 2015 (Figure 1).^21^ However, Peru's mCPR remains substantially below the regional average of 67% in South America.^2^ Use of contraception is much lower in the highland and jungle regions than the coastal region of the country, and lower among non-Spanish-speaking, indigenous women (Figure 4).^54^

The Seguro Integral de Salud (SIS) is a government-supported insurance scheme funded by general taxes that provides a basic package of services for those below the poverty line. It was implemented in 2007 and now covers 45% of women of reproductive age. It is autonomous from Peru's mandatory SHI scheme for formal sector workers and their dependents, EsSalud, which is funded by payroll taxes and covers 26% of women of reproductive age.^21^

SIS beneficiaries can obtain all major contraceptive methods free of charge from any Ministry of Health facility. Sterilization, however, is largely unattainable because of barriers put in place in response to allegations of forced sterilization in the late 1990s. EsSalud members also pay nothing OOP for family planning services, but EsSalud facilities provide a more limited range of methods. Private insurance plans do not consistently cover family planning services, and many EsSalud beneficiaries as well as clients with private insurance are funneled to free government clinics.^54^ As a result, these clinics reportedly face oversaturation, underfunding, and stock-outs. This implicit rationing of family planning services contributes to high OOP spending on family planning, which accounts for an estimated 70% of total family planning expenditures in Peru. OOP costs present a substantial barrier to family planning access for the poor, for whom family planning costs may represent a disproportionate burden. Other marginalized groups also face limited access to family planning, either because of geography, ethnicity, or age.^55^

Peru's private health insurance schemes often do not cover family planning, resulting in overcrowding and excess demand in public facilities.

Expansion of the family planning method mix provided through EsSalud and more complete coverage of the poor and near-poor by SIS may help to alleviate some of the pressure on public facilities. At the same time, Peru must pursue additional efforts to reach poor, indigenous, and otherwise marginalized women by increasing investment in health services in rural areas, adopting more culturally sensitive family planning practices, and rebuilding public confidence in family planning services.

## DISCUSSION

The country-level analysis revealed several major trends across our 4 areas of study.

### Family Planning Coverage

The 9 countries in our study demonstrated varying levels of family planning progress. The population-weighted average mCPR in our sample of countries is 60.9%, compared with 66.7% regionally,^2^ and it varied from 31.3% in Haiti (the lowest in the region) to 74.7% in Costa Rica (the highest in the group and among the highest in the region). However, over the past 10 years, the gap in mCPR across LAC has narrowed. In 5 of the 9 countries studied, mCPR increased substantially in the last decade, and this was predominantly in those countries with lower initial contraceptive prevalence (Figure 1). On the other hand, in some of the countries with higher initial mCPR, such as Costa Rica and the Dominican Republic, the mCPR has begun to plateau.

We found less variation in unmet need for family planning across the countries observed, with the exception of Haiti, which had an unmet need of 35.3%—more than twice that of the next highest country in our sample (Guatemala, 14.1%). Use of traditional methods was observed to be highest in countries with large indigenous populations, including Peru and Guatemala. In the latter, traditional methods accounted for 20% of the overall method mix in 2014.^36^ The percentage of total demand satisfied by modern family planning methods ranged from 45% in Haiti to 89% in Costa Rica.

Modern contraceptive use was lowest among the most vulnerable and marginalized populations, particularly indigenous, poor, and uninsured women. Indigenous populations across the 5 countries with available data had an mCPR that was on average 20% lower than the general population (Figure 4). Generally, countries with the largest indigenous populations reported the greatest inequities. Costa Rica and Colombia, with smaller indigenous populations, have achieved substantial success in the expansion of family planning. However, they still face some challenges in reaching their poor and indigenous populations. To address these inequities, many LAC countries are beginning to take steps to address sociocultural barriers to family planning uptake among indigenous women. Strategies have included using tailored demand creation strategies such as peer promotion, culturally appropriate communication and behavior change strategies, integration of family planning with water and sanitation projects, and cash transfer programs.^56^

Across countries reviewed, modern contraceptive use was lowest among the most vulnerable and marginalized populations, particularly indigenous, poor, and uninsured women.

The modern contraceptive method mix varied across countries in our sample in ways that may not always be representative of the LAC region (Figure 2). Female sterilization was the most common method within the sample, accounting for on average approximately 34% of family planning users, compared with the LAC average of 49%.^2^ Injectable contraception was the second most common method at 20% of users, compared with 3% regionally. In each country, the 2 most popular methods accounted for at least 60% of use, and in all countries except Colombia, the top 3 methods accounted for at least 75% of use. The method mix in the countries was thus slightly more concentrated than in the LAC region overall, where, on average, the top 2 and 3 methods accounted for 56% and 69%, respectively, of method mix. Notably, the countries with high insurance coverage and benefits that included all major modern contraceptive methods did not necessarily exhibit a more diverse method mix. For example, in Chile the top 2 methods accounted for 89% of the method mix. This may suggest that factors other than cost and availability of methods, such as information or cultural preferences, play an important role in determining method mix.

### Family Planning Financing

In recent years, financing for family planning programs has shifted from external to domestic sources, with most LAC countries having graduated from external donor funding.^1^^,^^57^ In 7 of the study countries—Haiti and Jamaica were excluded due to a lack of data—we estimate that approximately one-third of expenditures on family planning services, on average, came from government sources (Supplement 2), while public facilities accounted for more than half (55%) of the family planning methods provided. This should not be taken to mean that public facilities are more efficient and do more with less. There may be variation in method mix by source. For example, a majority of clients who seek sterilization services do so in public facilities, while condoms are most commonly purchased in private facilities. Public funds primarily finance services in government-operated facilities—either public or belonging to SHI programs. In Chile, Colombia, and the Dominican Republic, where private facilities are included within the SHI network and reimbursement for services is made by SHI schemes, the share of public family planning expenditures may be higher.

Despite the inclusion of family planning services in most countries' benefits packages, through either SHI or public facilities, OOP payments were a substantial portion of national family planning expenditures. In Guatemala, Honduras, and Peru, on average, 70% of family planning expenditures were private. This appears to be out of line with trends in THE. Among our sample of countries, OOP spending averaged less than one-third of THE; in 7 of the 9 countries, OOP spending was less than 35% of THE.^58^ (OOP as a share of THE was highest in Peru, at 52%.) Higher costs for family planning commodities and services in private facilities explain why OOP spending remains substantial, despite the fact that public facilities were cited as the primary source of family planning methods in population-level surveys for 8 of the 9 countries (Haiti was the exception). Even in countries where insurance coverage is high, including Chile, Costa Rica, and Colombia, continued high OOP spending suggests that there may be a need to examine insurance benefits in the context of family planning to understand if the included services match the methods demanded.

Although most countries' benefits packages included family planning, out-of-pocket spending remained a substantial part of total family planning expenditure.

### Health Financing

Financial protection from the cost of health care in the LAC region is typically available through 3 types of health financing modalities: SHI or other government-supported insurance, PHI, and tax-funded public health services, usually provided by the ministry of health. Eight of the 9 countries in our sample (all except Jamaica) have some form of SHI, financed primarily by contributions from formal sector employers and their employees through payroll taxes. In 5 countries—Chile, Colombia, Costa Rica, the Dominican Republic, and Peru—the SHI program includes a subsidized scheme that serves the poor and in some cases the non-poor workers in the informal sector (e.g., Chile's FONASA). In some of these countries with dual-track schemes (e.g., Chile and Colombia), enrollees in the contributory scheme had access to a broader range of health facilities, including the private sector, compared with enrollees in the subsidized scheme. However, in most countries, the packages of services in the SHI scheme have over time been equalized across all covered individuals, and usually include all major family planning methods. The SHI schemes in Guatemala, Haiti, and Honduras remain contributory only (i.e., limited to public and other formal sector employees who pay in). In these countries, less than 20% of the population is covered by an SHI scheme (Table).

8 of the 9 countries in our sample have some form of state-supported health insurance scheme.

Coverage through private insurers was limited within our sample. PHI coverage was less than 10% in 6 of the 9 countries; Chile was the only country to explicitly incorporate PHI schemes into its UHC agenda (with ISAPRES). Due to low total insurance coverage rates in some of our sampled countries, on average, nearly half of the people across the sample relied on public services or OOP payment for health care. Reliance on public health services or OOP spending was particularly high in Central America and the Caribbean. Although usually provided free of charge, public health services frequently face insufficient funding and stock-outs of essential medicines and supplies, which result in implicit rationing and poor quality of care.^16^^,^^48^^,^^59^ Many uninsured people, therefore, are forced to pay OOP for health services in private facilities or pharmacies. For the poor—who are also less likely to be enrolled in SHI due to informal employment or unemployment—these costs can cause substantial financial hardship.

### Family Planning Inclusion in UHC-Oriented Schemes

Our study found that family planning services have been relatively well-integrated into UHC-oriented schemes. However, variation exists across both countries and schemes (Figure 3). Eight of the 9 countries in the study provided public family planning services for free, typically in facilities operated by the ministry of health, typically catering to the uninsured. Only in Costa Rica were family planning services in public facilities limited to those enrolled in formal insurance schemes. In theory, the family planning services provided included all or most major contraceptive methods, yet not all facilities were equipped to provide all methods, and stock-outs were a major problem in many facilities and countries, particularly in low- and lower-middle-income countries. As a result, many uninsured clients were forced to pay OOP for family planning commodities in the private sector.

Family planning services have been relatively well-integrated into UHC-oriented schemes in LAC countries.

Coverage of family planning under government-supported SHI schemes varied substantially by countries' income level. In 5 of the 6 high- and upper-middle-income countries in our sample, the SHI schemes comprehensively covered family planning services (Jamaica was the exception). Generally, co-pays or user fees were not required. Some facilities did experience implicit rationing due to wait times, referrals, and sporadic stock-outs. In the 2 lower-middle-income countries in our sample, SHI schemes generally did not provide sufficient family planning coverage; they either provided a limited range of methods (Guatemala) or required clients to pay for FP fully OOP (Honduras) in scheme-linked facilities.

Family planning coverage varied substantially by countries' income level.

PHI played a relatively minor role in purchasing of family planning services, with Chile and Colombia being the only 2 countries in our sample where PHI regularly covered family planning. In other countries, sterilization was the only method regularly covered by private schemes.

Across our sample of countries, clients who paid OOP for contraceptive commodities faced costs ranging from $1 to more than $200 per couple-year of protection (personal communication, Karin Vrijburg, Researcher, NIDI, and Erik Beekink, Project leader, UNFPA-NIDI Resource Flows Project on Family Planning, NIDI, June, 2016). Permanent contraceptive procedures (i.e., sterilization) and long-acting reversible contraceptive methods, such as IUDs, tended to have the lowest costs per couple-year of protection but often carried large up-front OOP costs due to the need for facility-based services. In some countries, facility-based services, such as sterilization, had a one-time cost of more than $300, which presents a substantial financial barrier to poor clients. Data on the OOP cost of implants were lacking, which may present a lower-cost alternative to other long-acting methods. However, implants accounted for, on average, 2% of contraceptive method use in our sample of counties, and just 0.2% regionally. High OOP costs overall highlight the importance of financial protection for family planning for the poorest clients as well as the potential value of SHI and other UHC-oriented schemes in improving access and equity in family planning programs.

Our analysis does not attempt to draw a causal link between coverage status under UHC-oriented schemes and family planning use. We found that enrollment in government-supported insurance schemes (rather than reliance on public provision through ministry of health facilities), and particularly in SHI schemes, was associated with improved access to and uptake of modern family planning methods. In the 4 countries where population-level data were available, insured women, on average, had a mCPR 5.1 percentage points higher than uninsured women. The relationship between insurance coverage and family planning use appeared to be even more pronounced among the most financially vulnerable women (Figure 5). Among the poorest quintile of women, insured women had an mCPR 16.5 percentage points higher than those that were uninsured. Unsurprisingly, among uninsured women, the average mCPR in the highest income quintile was 10.5 percentage points higher than in the lowest. Paradoxically, among insured women, mCPR was actually higher on average—by 2.7 percentage points—among the poorest women than the wealthiest (Figure 5). In some cases, such as in the Dominican Republic, this may be due to the failure of PHI plans, which typically cover wealthier beneficiaries, to include family planning in their benefits package.

Enrollment in government-supported insurance schemes (rather than reliance on public health facilities) was associated with improved access to and uptake of modern family planning methods.

Among the poorest quintile of women, insured women had a modern contraceptive prevalence rate16.5 percentage points higher than those that were uninsured.

## CONCLUSIONS AND RECOMMENDATIONS

In the sample of high- and upper-middle-income LAC countries we studied, SHI and other government-supported health insurance schemes have been the major conduit for implementation of UHC goals. These schemes have been, by and large, successful in integrating family planning services into their benefits packages. In sampled low- and lower-middle-income countries, where the SHI schemes remain confined to public and formal sector employees, inclusion of family planning services as benefits has been limited and sporadic. Family planning users in low- and lower-middle-income countries remain primarily dependent on public facilities and OOP payment for family planning services. As a result, poor and marginalized clients often continue to face financial hardship when seeking family planning services.

SHI and allied insurance schemes do present a pathway for further improvements in family planning access and use, alongside ensuring sustainable financing of family planning programs in LAC. Particularly in lower-middle-income countries, expansion of SHI beyond the formal sector must also be accompanied by the explicit inclusion of comprehensive family planning services within the benefits package. At the same time, although PHI covers only a small portion of the population in most LAC countries, better family planning coverage (i.e., inclusion in benefits packages and expanded choice of methods) by these schemes can help to diversify choice and provide options for non-poor informal-sector workers who must otherwise seek services in potentially overwhelmed public facilities. Countries will need to take a comprehensive view of UHC—not only as SHI-led but also as encompassing both public provision and private insurance—to achieve sustainable financing of family planning.

Better inclusion of family planning in private health insurance schemes can help to diversify method choice and provide options for informal-sector workers.

Albeit with caveats, the broad success of several LAC countries in simultaneously scaling up both family planning and SHI programs can serve as an example to other countries, both within and outside the region, as they consider including equitable family planning access in their UHC goals. However, SHI and other government-supported schemes are not a panacea for increasing family planning access and ensuring sustainable family planning financing. Our analysis does not infer any causal relationship between increase in health insurance coverage and improved access to family planning or increased use of modern methods. However, this study suggests that for insurance schemes—public and private and other (i.e., provision of services by the ministry of health)—to positively impact family planning uptake and sustainability, deliberate and explicit steps must be taken to ensure family planning is included in these schemes.

Deliberate and explicit steps must be taken to ensure family planning is included in UHC-oriented schemes.

For a relationship between insurance coverage and family planning access to exist, insurance schemes must (1) target poor and informal sector populations, for whom OOP spending on family planning presents a substantial financial barrier; (2) include family planning, either explicitly or implicitly, in the covered package of services; (3) ensure sufficient human resources and commodities to prevent stock-outs and implicit rationing of family planning services (if services and commodities are provided through the public sector), or ensure that reimbursements for family planning services include commodities (if commodities are purchased by providers) and are sufficient to ensure availability of both services and method choice; and (4) reduce nonfinancial barriers to access, including those due to geography, cultural factors, service quality, and range of methods, to ensure that couples can use their insurance to access their preferred contraceptive method. In many countries, scale-up of insurance schemes will coexist with government provision of services, especially to the poor, through public facilities. In those contexts, it will also be critical to ensure that enrollees in formal insurance schemes are not funneled to public facilities, causing crowding out of services for the uninsured.

When those covered by insurance are sent to public facilities for family planning methods, overcrowding leads to lack of access for the uninsured.

A limitation of our study, driven by resources, is that we did not look closely at variations in purchasing methods for family planning services (e.g., whether family planning was included under capitation or was purchased fee-for-service) under SHI and other government-supported schemes. Purchasing methods have influence on provider incentives and can affect access to and inclusion of family planning services.

This analysis constitutes a first step in associating family planning with UHC progress, particularly in LAC. Financing-related analyses are only part of the set of studies required to situate sustainable family planning programs within the context of UHC, particularly as many countries continue to transition from external to domestic funding sources. While many LAC countries have adopted a view of health as a fundamental right of citizens and have allocated funds to ensure access to services for poor and vulnerable populations, there remain unique challenges that continue to limit access to family planning. Full implementation of a rights-based approach to health will require nuanced strategies for including family planning in SHI and other UHC-oriented schemes. Although countries beyond LAC can learn lessons from the overall success and limitation of family planning-UHC linkages in the region, LAC countries with lagging family planning indicators can also apply these lessons to address gaps in family planning inclusion in their own SHI and UHC-oriented schemes.

Unique challenges remain in LAC countries to improve access to family planning.

## Supplementary Material

Supplement 1

## Notes

**Peer Reviewed**

**First Published Online:** 2017 July 31

## References

[1] Bertrand J Ward V Santiso-Gálvez R Family planning in Latin America and the Caribbean: the achievements of 50 years. Chapel Hill, NC: MEASURE Evaluation; 2015 https://www.measureevaluation.org/resources/publications/tr-15-101 Accessed July 10, 2017.

[2] United Nations (UN), Department of Economic and Social Affairs, Population Division. Trends in contraceptive use worldwide 2015. New York: UN; 2015 http://www.un.org/en/development/desa/population/publications/pdf/family/trendsContraceptiveUse2015Report.pdf Accessed July 10, 2017.

[3] Dmytraczenko T Almeida G Toward universal health coverage and equity in Latin America and the Caribbean: evidence from selected countries. Washington, DC: World Bank; 2015 https://openknowledge.worldbank.org/bitstream/handle/10986/22026/9781464804540.pdf?sequence= Accessed July 10, 2017.

[4] What is universal coverage? World Health Organization website. http://www.who.int/health_financing/universal_coverage_definition/en/ Accessed July 10, 2017.

[5] Giedion U Bitrán R Tristao I, editors. Health benefit plans in Latin America: a regional comparison. Washington, DC: Inter-American Development Bank, Social Protection and Health Division; 2014 https://publications.iadb.org/handle/11319/6484?locale-attribute=en Accessed July 10, 2017.

[6] Atun R de Andrade LO Almeida G., Health-system reform and universal health coverage in Latin America. Lancet. 2015; 385(9974):1230–1247. 10.1016/S0140-6736(14)61646-9. 25458725

[7] Technical issue briefs: Latin America and the Caribbean. United States Agency for International Development website. https://www.usaid.gov/what-we-do/global-health/family-planning/family-planning-resources/issue-briefs-latin-america-caribbean. Last updated September 6, 2016 Accessed July 10, 2017.

[8] Naik R Morgan L Wright J The role of health insurance in family planning. Washington, DC: Population Reference Bureau; 2015 http://www.prb.org/Publications/Reports/2015/family-planning-health-insurance.aspx Accessed July 10, 2017.

[9] Instituto Nacional de Estadísticas. Estadísticas vitales, anuario 2013. Santiago, Chile: Instituto Nacional de Estadísticas; 2015.

[10] United Nations (UN), Department of Economic and Social Affairs, Population Division. Model-based estimates and projections of family planning indicators 2015. New York: UN; 2015 http://www.un.org/en/development/desa/population/theme/family-planning/cp_model.shtml Accessed July 10, 2017.

[11] Superintendencia de Seguridad Social (SSS). Estadísticas de seguridad social 2015. Santiago, Chile: SSS; 2016 http://www.suseso.cl/boletin-estadistico/ Accessed July 10, 2017.

[12] Ministry of Health and Social Protection (MINSALUD). Informe 2014–2015, sector administrativo de salud y protección social al honorable Congreso de la República. Bogota, Colombia: MINSALUD; 2015 http://studylib.es/doc/4545570/informe-al-congreso-2014–2015—ministerio-de-salud-y-pro Accessed July 10, 2017.

[13] Caja Costarricense de Seguro Social (CCSS). Indicadores de la seguridad social: 2008–2013. San Jose, Costa Rica: CCSS; 2014 http://www.ccss.sa.cr/est_demografica Accessed July 10, 2017.

[14] Ministry of Public Health (MSP); Centro de Estudios Sociales y Demográficos; ICF International. Encuesta demográfica y de salud 2013. Santo Domingo, Dominican Republic: MSP; 2014 http://dhsprogram.com/pubs/pdf/PR43/PR43.pdf Accessed July 10, 2017.

[15] Instituto Guatemalteco de Seguridad Social (IGSS). Boletín estadístico afiliación 2014. Guatemala City: IGSS; 2015 http://www.igssgt.org/images/informes/subgerencias/boletin_estadistico_afiliacion2014.pdf Accessed July 10, 2017.

[16] Avila C Bright R Gutierrez JC Hoadley K Manuel C Romero N Guatemala health system assessment. Bethesda, MD: Health Finance & Governance Project, Abt Associates Inc; 2015 https://www.usaid.gov/documents/1862/guatemala-health-system-assessment-2015 Accessed July 10, 2017.

[17] Health Policy Project (HPP). Health financing profile: Haiti. Washington, DC: HPP; 2016 http://www.healthpolicyproject.com/pubs/7887/Haiti_HFP.pdf Accessed July 10, 2017.

[18] Instituto Hondureño de Seguridad Social (IHSS). IHSS en cifras: la serie: 2004–2015. Tegucigalpa, Honduras: IHSS; 2015 http://ihss.hn/?page_id=738 Accessed July 10, 2017.

[19] White C 10 years of the NHF: performance challenges and lessons of experience. Presented at: 8th Caribbean Conference on Health Financing Initiatives; November 12–14, 2013; Montego Bay, Jamaica.

[20] Wilks R Younger N Tulloch-Reid M McFarlane S Francis D Jamaica health and lifestyle survey 2007–8. Mona, Jamaica: University of the West Indies; 2008 http://moh.gov.jm/data/jamaica-health-and-lifestyle-survey-2007-8/ Accessed July 10, 2017.

[21] Instituto Nacional de Estadística e Informática (INEI). Perú: encuesta demográfica y de salud familiar 2015: nacional y departamental. Lima, Peru: INEI; 2016 https://www.inei.gob.pe/media/MenuRecursivo/publicaciones_digitales/Est/Lib1211/pdf/Libro.pdf Accessed July 10, 2017.

[22] Class D Cavagnero E Sunil Rajkumar AS Ferl K Health financing profile–Chile. Washington, DC: World Bank; 2013 http://documents.worldbank.org/curated/en/726041468238167664/Chile-Health-financing-profile Accessed July 10, 2017.

[23] Health Policy Plus. Financing family planning: Chile. Washington, DC: Health Policy Plus; 2016 http://www.healthpolicyplus.com/ns/pubs/2068-2103_HPSFIBriefChileEnglish.pdf Accessed July 10, 2017.

[24] Ojeda G Ordóñez M Ochoa LH Encuesta nacional de demografía y salud 2010. Bogota, Colombia: Profamilia; 2011 https://dhsprogram.com/pubs/pdf/FR246/FR246.pdf Accessed July 10, 2017.

[25] Dutta A Hongoro C Scaling up national health insurance in Nigeria: learning from case studies of India, Colombia, and Thailand. Washington, DC: Futures Group, Health Policy Project; 2013 http://www.healthpolicyproject.com/index.cfm?id=publications&get=pubID&pubID=96 Accessed July 10, 2017.

[26] Plan obligatorio de salud. Ministry of Health and Social Protection (MINSALUD) [Colombia] website. https://www.minsalud.gov.co/salud/POS/Paginas/plan-obligatorio-de-salud-pos.aspx Accessed July 10, 2017.

[27] Montenegro Torres F Bernal Acevedo O Colombia case study: the subsidized regime of Colombia's national health insurance system (UNICO). Washington, DC: World Bank; 2013 http://documents.worldbank.org/curated/en/727721468239997995/pdf/749610NWP0COLO00Box374316B00PUBLIC0.pdf Accessed July 10, 2017.

[28] Ministry of Health and Social Protection (MINSALUD). Resolución 1552 de 2013. Bogota, Colombia: MINSALUD; 2013 http://www.alcaldiabogota.gov.co/sisjur/normas/Norma1.jsp?i=53131 Accessed July 10, 2017.

[29] Health Policy Plus. Financing family planning: Colombia. Washington, DC: Health Policy Plus; 2016 http://www.healthpolicyplus.com/ns/pubs/2068-2105_HPSFIBriefColombiaEnglish.pdf Accessed July 10, 2017.

[30] Ministry of Health; UNICEF. Encuesta de indicadores múltiples por conglomerados 2011. San Jose, Costa Rica: Ministry of Health; 2013 https://www.unicef.org/costarica/docs/cr_pub_MICS_2011.pdf Accessed July 10, 2017.

[31] Siu M CCSS rechaza atención médica de no asegurados, a menos que vida esté en riesgo o paguen. crhoy.com. 7 23, 2013 http://www.crhoy.com/ccss-rechaza-atencion-medica-de-no-asegurados-a-menos-que-vida-este-en-riesgo-o-paguen/ Accessed July 10, 2017.

[32] United States Agency for International Development (USAID). USAID'S partnership with Costa Rica advances family planning. Washington, DC: USAID; 2016 https://www.usaid.gov/sites/default/files/documents/1864/Costa-Rica-508.pdf Accessed July 10, 2017.

[33] Sáenz MdelR Acosta M Muiser J Bermúdez JL. Sistema de salud de Costa Rica. Salud Publica Mex. 2011; 53(suppl 2):s156–s167. 21877081

[34] Métodos anticonceptivos ofrecidos por la seguridad social en Costa Rica. Salud para todos del Dr. E-Salud Blog website. 4 4, 2013 http://doctoresalud.blogspot.com/2013/04/metodos-anticonceptivos-ofrecidos-por.html Accessed July 10, 2017.

[35] Montenegro Torres F Costa Rica case study: primary health care achievements and challenges within the framework of the social health insurance. Washington, DC: World Bank; 2013 http://documents.worldbank.org/curated/en/991581468233939710/pdf/749620NWP0COST00Box374316B00PUBLIC0.pdf Accessed July 10, 2017.

[36] United States Agency for International Development (USAID). USAID's partnership with the Dominican Republic advances family planning. Washington, DC: USAID; 2016 https://www.usaid.gov/what-we-do/global-health/family-planning/countries/dominican-republic/issue-briefs Accessed July 10, 2017.

[37] Health Policy Plus. Financing family planning: Dominican Republic. Washington, DC: Health Policy Plus; 2016 http://www.healthpolicyplus.com/ns/pubs/2068-2116_HPSFIBriefDominicanRepublicEnglish.pdf Accessed July 10, 2017.

[38] Ministry of Public Health and Social Assistance (MSPAS); Instituto Nacional de Estadística (INE); ICF International. Encuesta nacional de salud materno infantil 2014–2015: informe de indicadores básicos. Guatemala City: MSPAS; 2015 http://pdf.usaid.gov/pdf_docs/PBAAD728.pdf Accessed July 10, 2017.

[39] Lao Pena C Improving access to health care services through the Expansion of Coverage Program (PEC): the case of Guatemala. Washington, DC: World Bank; 2013 http://documents.worldbank.org/curated/en/967411468039876158/pdf/750010NWP0Box30ge0Program0GUATEMALA.pdf Accessed July 10, 2017.

[40] Health Policy Plus. Financing family planning: Guatemala. Washington, DC: Health Policy Plus; 2016 http://www.healthpolicyplus.com/ns/pubs/2068-2111_HPSFIBriefGuatemalaEnglish.pdf Accessed July 10, 2017.

[41] Ministère de la Santé Publique et de la Population à Haïti (MSPP). Enquête mortalité, morbidité et utilisation des services EMMUS-V. Port-au-Prince, Haiti: MSPP; 2012 http://www.dhsprogram.com/publications/publication-FR273-DHS-Final-Reports.cfm Accessed July 10, 2017.

[42] Health Policy Project (HPP). Health financing profile: Haiti. Washington, DC: Palladium, HPP; 2015 www.healthpolicyproject.com/pubs/7887/Haiti_HFP.pdf Accessed July 10, 2017.

[43] Ministère de la Santé Publique et de la Population (MSPP). Enquête sur les produits et les services de santé reproductive dans 132 institutions sanitaires des 10 départements d'Haïti. Tabarre, Haiti: MSPP; 2015 https://mspp.gouv.ht/site/downloads/enquete%20DSF%202015.pdf Accessed July 10, 2017.

[44] Dieuve J Pierre PR Pierre-Victor M Stiven M Amélioration de la qualité des soins a la maternité de l'hôpital de l'OFATMA. Port-au-Prince, Haiti: Programme de formation en management et gestion des services de santé en Haïti (DESS MGSS); 2004 http://www.dess.fmp.ueh.edu.ht/pdf/groupe_OFATMA.pdf Accessed July 10, 2017.

[45] Berrand J Santiso-Galvez R Ward V La planification familiale en Haïti: Les accomplissements des 50 dernières années. Chapel Hill, NC: Measure Evaluation; 2015 https://www.measureevaluation.org/resources/publications/sr-15-118h-fr Accessed July 10, 2017.

[46] Le Président de la République inaugure le département de physiothérapie de l'OFATMA et lance la Carte Rose d'assurance santé. 5 28, 2012 https://www.facebook.com/notes/michel-martelly/le-pr%C3%A9sident-de-la-r%C3%A9publique-inaugure-le-d%C3%A9partement-de-physioth%C3%A9rapie-de-lofat/371976482851440/ Accessed July 10, 2017.

[47] Secretaría de Salud (SS); Instituto Nacional de Estadística (INE); ICF International. Encuesta nacional de demografía y salud 2011–2012. Tegucigalpa, Honduras: SS, INE, and ICF International; 2013 http://dhsprogram.com/publications/publication-FR274-DHS-Final-Reports.cfm Accessed July 10, 2017.

[48] Anigstein C Lenci S Tobar F Consultoría para la realización de un análisis explicativo de las razones que determinan desabastecimiento de anticonceptivos en América Latina y el Caribe (LAC). New York: United Nations Population Fund; 2015 http://www.redhum.org/uploads/documentos/pdf/Redhum-Ec-TorConsultorStockout-UNFPALAC-20150515-20150519-MP-16467.pdf Accessed July 10, 2017.

[49] United States Agency for International Development (USAID). USAID'S partnership with Honduras advances family planning. Washington, DC: USAID; 2016 https://www.usaid.gov/sites/default/files/documents/1864/honduras-508.pdf Accessed July 10, 2017.

[50] National Family Planning Board (NFPB). Annual family planning statistical report 2010. Kingston, Jamaica: NFPB; 2012 http://jnfpb.org/assets/Annual-Family-Planning-Statistical-Report-2010.pdf Accessed July 10, 2017.

[51] Health Policy Plus. Financing family planning: Jamaica. Washington, DC: Health Policy Plus; 2016 http://www.healthpolicyplus.com/ns/pubs/2068-2113_HPSFIBriefJamaicaEnglish.pdf Accessed July 10, 2017.

[52] Mziray E Haacker M Chao S Assessing the financial sustainability of Jamaica's HIV program. Washington, DC: World Bank; 2012 http://documents.worldbank.org/curated/en/447451468262808471/Assessing-the-financial-sustainability-of-Jamaicas-HIV-Program Accessed July 10, 2017.

[53] Chao S Jamaica's effort in improving universal access within fiscal constraints. Universal Health Coverage Studies Series (UNICO). Washington, DC: World Bank; 2013 http://documents.worldbank.org/curated/en/408381468044133381/Jamaicas-effort-in-improving-universal-access-within-fiscal-constraints Accessed July 10, 2017.

[54] Class D Cavagnero E Sunil Rajkumar A Ferl K Health financing profile–Peru. Washington, DC: World Bank; 2013 http://documents.worldbank.org/curated/en/242851468099278957/Peru-Health-financing-profile Accessed July 10, 2017.

[55] El Minsa activa cerco epidemiológico para evitar propagación del zika en Jaén, Yurimaguas, Zarumilla, Tocache y Pucallpa. Ministry of Health (MINSA) [Peru] website. http://www.minsa.gob.pe/?op=51&nota=18545. June 6, 2016 Accessed July 10, 2017.

[56] Kaufman H Addressing sociocultural barriers to family planning among indigenous populations in Latin America. Presented at: Implementing Best Practices Initiative Regional Meeting; June 14–16, 2016; Lima, Peru.

[57] Shen AK Farrell MM Vandenbrouke MF Fox E Pablos-Mendez A. Applying lessons learned from the USAID family planning graduation experience to the GAVI graduation process. Health Policy Plan. 2015; 30(6):687–695. 10.1093/heapol/czu045. 24974106

[58] Global Health Expenditure Database. Geneva: World Health Organization http://www.who.int/health-accounts/ghed/en/. Updated in March every year Accessed July 10, 2017.

[59] Barden-O'Fallon J Availability and quality of FP services in faith-based organizations: a three country study. Presented at: International Conference on Family Planning; January 26, 2016; Nusa Dua, Indonesia.

