# Supplementary material for: Family Planning in the Context of Latin America's Universal Health Coverage Agenda
Source: Glob Health Sci Pract. 2017 Sep 27;5(3):382–98. doi: 10.9745/GHSP-D-17-00057 (PMC5620336; doi:10.9745/GHSP-D-17-00057)
Supplement: Supplement 1 [file GHSP-D-17-00057_index.html]

Supplement to Family Planning in the Context of Latin America’s Universal Health Coverage Agenda | Global Health: Science and Practice

## Supplemental material

- Text s01, PDF - Text s01, PDF
- Text s02, PDF - Text s02, PDF
