# Supplementary material for: Family Planning in the Context of Latin America's Universal Health Coverage Agenda
Source: Glob Health Sci Pract. 2017 Sep 27;5(3):382–98. doi: 10.9745/GHSP-D-17-00057 (PMC5620336; doi:10.9745/GHSP-D-17-00057)
Supplement: Supplement 1 [file 17-00057-Dutta-Supplement1.pdf]

Fagan T, Dutta A, Rosen J, Olivetti A, Klein K. Family planning in the context of Latin America's universal health coverage agenda. Glob Health Sci Pract. 2017;5(3). https://doi.org/10.9745/GHSP-D-17-00057

SUPPLEMENT 1: Indicator List

No.	Indicator
**Family Planning Coverage**	
1	Current method mix: % for each method, married women and all women
2	Projected method mix (to 2020): % for each method, all women
3	Current contraceptive prevalence rate, modern methods (mCPR): married women and all women
4	Projected mCPR (to 2020): all women
5	Current unmet need: married women and all women
6	Projected unmet need (to 2020): all women
7	mCPR by income or wealth quintile, latest year
8	mCPR by geographic area, latest year
9	mCPR by ethnic group or indigenous/nonindigenous identity, latest year
10	Total fertility rate, latest year
**Family Planning Financing**	
11	Percentage of all FP commodities funded by source (government, households/OOP, insurers, external sources—specify by donor)
12	Estimated split (%) of in-country procurement and supply chain management costs by source (government, donor, other)
13	Estimated contribution (%) from external sources to FP service delivery (HRH, demand creation, other non-PSM)
14	Estimated financing gap for FP by year (to 2020), beginning from latest year available
15	Percentage of FP services (supply + information) for current users received by sector (public, private-not-for-profit, private-for-profit)
**Health Financing**	
16	National Health Accounts disaggregation of total health expenditure (THE) by source, latest year available from in-country report
17	THE per capita, latest available from in-country report or calculated

18 National health insurance coverage (% of population) by insurance scheme (social, community, national, private)

19 Health insurance coverage (% of population) for key government-supported schemes, by wealth quintile

20 Health insurance coverage (% of population) for key government-supported schemes, by geographic area [aligned with indicator #8]

21 User fees policy in public sector facilities (universally exempt; means-based exemption or waiver; other), by primary vs. hospital level

22 Approximate user fee (US$) for 1 outpatient visit, relevant to FP method service delivery, in 2016

23 Catastrophic health expenditure incidence, latest year available [exact definition will vary by country-specific data reported]

24 Government allocation to health (with on-budget support from donors) as % of total government expenditure, latest fiscal year

25 Major health insurance scheme #1: *characteristics* (e.g., number of members and beneficiaries, purchasing modality, type of providers)

26 Major health insurance scheme #2: *characteristics* (e.g., number of members and beneficiaries, purchasing modality, type of providers)

27 Major health insurance scheme #1: *contributions* (e.g., payroll-based taxes and % contributed by employer and/or employee, government subsidies, other)

28 Major health insurance scheme #2: *contributions* (e.g., payroll-based taxes and % contributed by employer and/or employee, government subsidies, other)

29 Major health insurance scheme #1: *benefits package* (e.g., characteristics of coverage: up to secondary or tertiary; based on a positive list, negative list or both; inclusion of preventive and promotive health care)

30 Major health insurance scheme #2: *benefits package* (e.g., characteristics of coverage: up to secondary or tertiary; based on a positive list, negative list or both; inclusion of preventive and promotive health care)

Family Planning Inclusion in UHC-Oriented Schemes

31 Status of inclusion of modern contraceptive methods in major health insurance scheme #1 (included, included with caveats [specify], not included)

32 Status of inclusion of modern contraceptive methods in major health insurance scheme #2 (included, included with caveats [specify], not included)

33 Co-pay or other user fee for accessing FP under major health insurance scheme #1, by method if so disaggregated; specifics for tubal ligation and vasectomy

34 Co-pay or other user fee for accessing FP under major health insurance scheme #2, by method if so disaggregated; specifics for tubal ligation and vasectomy

Fagan T, Dutta A, Rosen J, Olivetti A, Klein K. Family planning in the context of Latin America's universal health coverage agenda. Glob Health Sci Pract. 2017;5(3). https://doi.org/10.9745/GHSP-D-17-00057

35	Cost characteristics of FP services for uninsured population accessing care from public sector (free, partially subsidized, unsubsidized [must purchase OOP])
36	Specific modern methods included/excluded from public sector provision (for uninsured population): list of methods
37	Methods available with implicit or explicit subsidy in retail outlets due to social marketing, and level of subsidy vs. equivalent unsubsidized product

Abbreviations: FP, family planning; HRH, human resources for health; mCPR, modern contraceptive prevalence rate; OOP, out of pocket; PSM, Procurement and Supply Management; THE, total health expenditure; UHC, universal health coverage.
