# Supplementary material for: Family Planning in the Context of Latin America's Universal Health Coverage Agenda
Source: Glob Health Sci Pract. 2017 Sep 27;5(3):382–98. doi: 10.9745/GHSP-D-17-00057 (PMC5620336; doi:10.9745/GHSP-D-17-00057)
Supplement: Supplement 1 [file 17-00057-Dutta-Supplement2.pdf]

Fagan T, Dutta A, Rosen J, Olivetti A, Klein K. Family planning in the context of Latin America's universal health coverage agenda. Glob Health Sci Pract. 2017;5(3). https://doi.org/10.9745/GHSP-D-17-00057

SUPPLEMENT 2: Estimates of Family Planning Expenditures

We estimated the shares of public and private sector funding for family planning (FP) in each of the selected countries using a variety of existing data sources (see Table, next page). First, we projected contraceptive prevalence and method mix for all women using trend analysis of past population-level surveys (Multiple Indicator Cluster Surveys, Demographic Health Surveys [DHS], or similar national surveys). Then the total number of users of each contraceptive method was calculated for 2015 by multiplying the contraceptive prevalence rate and method mix by the number of women of reproductive age, taken from the *World Population Prospects: The 2015 Revision*.[1] These totals were disaggregated by method source, using DHS estimates. Where DHS data were unavailable, we used aggregated data, provided by the Netherlands Interdisciplinary Demographic Institute (NIDI) (personal communication, Karin Vrijburg, Researcher, NIDI, and Erik Beekink, Project leader, UNFPA-NIDI Resource Flows Project on Family Planning, NIDI, June, 2016), on the source (i.e., public or private facilities) of all contraceptive methods by country. Lastly, the total number of women using each method, by source, was multiplied by country-specific unit cost estimates for both the public and private sector. Public-sector estimates were taken from estimates by Eva Weismann (personal communication, Eva Weissman, independent consultant, 2014). Private-sector estimates were provided by NIDI (personal communication, Karin Vrijburg, Researcher, NIDI, and Erik Beekink, Project leader, UNFPA-NIDI Resource Flows Project on Family Planning, NIDI, June, 2016), although the study team made two adjustments to the annualized estimate of the cost of condoms: The assumed number of condoms per client per year was changed from 100 to 120 for all countries. For Peru, the total cost per year was changed from $291 to $28 based on input from in-country experts. Total costs by method were then summed for both the public and private sectors to calculate the relative shares of FP financing. The results of these calculations can be found in the relevant Health Policy Plus country briefs.[2]

This methodology faces some limitations. Most significantly, the use of DHS estimate of method source as a proxy for financing source fails to capture reimbursement of private facilities through public health insurance schemes. This concern is most relevant in countries such as Colombia and the Dominican Republic, where public schemes regularly include service provision through private facilities. On the other hand, in countries such as Guatemala and Peru, where social health insurance networks include *only* public facilities, the DHS method source estimate should be a highly accurate proxy. When NIDI data on method source were used in place of DHS data, estimates of private expenditures likely fail to capture the contribution of private health insurance. However, in most countries, with the exception of Chile, private insurance coverage is low and unlikely to affect estimates significantly. More importantly, NIDI estimates do not disaggregate source by method. High costs per protection-year for shorter-acting methods, particularly condoms, which are primarily obtained in private facilities, may lead to underestimates of the private contribution to FP expenditures.

Table: Estimated Family Planning Expenditures (US$) by Source (Public or Private), 2015

	Public (Including SHI-Associated Facilities)		Private	
Chile	$9,504,118	46%	$11,351,863	54%
Colombia	$73,574,606	18%	$334,189,430	82%
Costa Rica	$13,393,967	38%	$21,743,680	62%
Dominican Republic	$8,584,881	40%	$12,660,971	60%
Guatemala	$10,702,166	37%	$18,342,870	63%
Honduras	$12,242,996	23%	$41,930,243	77%
Peru	$18,863,172	30%	$44,742,559	70%

Abbreviation: SHI, social health insurance.

References

1. United Nations (UN), Department of Economic and Social Affairs, Population Division. World population prospects: the 2015 revision. New York: UN; 2015. Available from: https://esa.un.org/unpd/wpp/
2. Multiple country-specific publications by Health Policy Plus. For example: Financing family planning: Chile. Washington, DC: Palladium; 2016. Also: Financing family planning: Colombia. Washington, DC: Palladium; 2016. Publications available at: http://www.healthpolicyplus.com/pubs.cfm?get=2068
